# Interventions for reducing unplanned paediatric admissions: an observational study in one hospital

**DOI:** 10.1136/bmjpo-2017-000235

**Published:** 2018-03-28

**Authors:** Kerryn Husk, Vashti Berry, Richard Tozer, Gina Skipwith, Robert Radmore, Susan Ball, Obioha C Ukoumunne, Stuart Logan

**Affiliations:** 1 NIHR CLAHRC South West Peninsula (PenCLAHRC), Faculty of Medicine and Dentistry, University of Plymouth, Plymouth, UK; 2 NIHR CLAHRC South West Peninsula (PenCLAHRC), University of Exeter Medical School, University of Exeter, Exeter, UK; 3 Torbay and South Devon NHS Foundation Trust, Torbay Hospital, Torquay, UK

**Keywords:** health services research, general paediatrics

## Abstract

**Objective:**

Evidence on how best to intervene to improve paediatric acute care and therefore reduce unplanned hospital admissions is weak. We describe service evaluation work at one hospital to assess interventions at critical clinical and service decision points.

**Design:**

We conducted an observational study using routine daily-collected data (April 2009–December 2015) from a medium-sized district general hospital in south-west UK, using before-and-after comparisons of admissions-related data to evaluate two interventions implemented in April and November 2014, respectively: (1) an advice and guidance (A&G) phone line, where a senior paediatrician is available for general practitioners (GPs) and emergency department (ED) and (2) a Short Stay Paediatric Assessment Unit (SSPAU). We analysed data on all admitted children (<18 years) in the catchment area (population estimate 27 740 in 2015). Outcomes were GP-referred attendances, ward admissions, less than 1 day admissions and length of stay.

**Results:**

A&G phone line was associated with a reduction in the mean number of less than 1 day admissions per month (difference in means before and after intervention −16.6 (95% CI −0.2 to −32.9)) and an increase in overall monthly bed-days (difference 72.5 (95% CI 21.0 to 124.0)), but there was little evidence of a change in GP-referred attendances or ward admissions. SSPAU was associated with a reduction in the mean number of monthly ward admissions (difference −34.6 (95% CI –21.3 to −48.0)) and less than 1 day admissions (difference in means −21.7 (95% CI −8.4 to −35.1)) and a reduction in the mean number of overall bed-days per month (difference −50.2 (95% CI −12.1 to −88.3)).

**Conclusions:**

Interventions for reducing time taken to senior clinician review may be effective in better managing paediatric acute care. Further work should explore results by age, condition and injury/illness status.

**What is already known on this topic?**

- Paediatric admissions are rising year by year in the UK.
- Evidence for interventions to better manage paediatric acute care and therefore reduce avoidable admissions is lacking.

**What this study hopes to add?**

- In a single hospital, an advice and guidance phone line was associated with fewer less than 1 day admissions, but an increase in overall bed-days.
- Short stay paediatric assessment unit (SSPAU) was associated with a reduction in ward admissions, less than 1 day admissions and overall bed-days.
- There are indications that advice and guidance and SSPAU, as examples of interventions reducing time taken to senior clinician review, are effective in better managing paediatric acute care.

## Introduction

### Background

Avoiding excess unplanned admissions is a UK National Health Service priority, with acute paediatric admissions rising year by year since 2003.^1 2^ While rates vary by area (and indeed in the site included in this study admission rates are flat),^3^ such increases are unsustainable and remain a research priority.^4 5^ Admission to hospital is an undesirable outcome for children and their parents for many reasons, including disruption to family life, increased emotional distress and exposure to infections. There are also significant cost implications of a hospital admission. The six most common conditions resulting in the presentation for paediatric acute care are the ‘big 6’ conditions: bronchiolitis/croup, fever, gastroenteritis, head injury, wheezy child/asthma and abdominal pain.^6 7^

While not well understood, the reasons for increased admissions are likely to be linked to changes in primary care provision, risk aversion among junior clinicians, a ‘defensive model’ of admission, advances in care reducing length of stay, funding arrangements and reduced parental experience in dealing with childhood illness.^8^ What constitutes a hospital ‘admission’ has also changed.^9–13^ Coon *et al* 9 examined the evidence for interventions intended to reduce acute paediatric admissions, including trials examining the effectiveness of five common initiatives: (1) consultant versus trainee decision on admission, (2) consultant telephone triage, (3) short stay/observation/assessment units, (4) algorithm-based care at admission and (5) next-day paediatric clinics. The evidence identified was weak and results equivocal; no firm conclusions could be drawn on effective initiatives for reducing admissions while avoiding negative impacts on those discharged. However, many hospitals are trying to change the organisation of care based on existing evidence and clinical experience.

The Royal Devon and Exeter Hospital worked with the NIHR CLAHRC South West Peninsula (PenCLAHRC) and the South West Strategic Clinical Network (SWSCN) to implement an evidence-driven ‘best guess’ change in paediatric service delivery. This comprised the establishment of a short stay paediatric assessment unit (SSPAU), the design of which is largely derived from adult clinical decision units.^14 15^ This change was associated with an 18% fall in the number of overnight admissions in 2013 compared with the preceding 4-year period 2009–2012 (Martin *et al* submitted).

As a result, the SWSCN partnered with PenCLAHRC to build a broader evidence base by mapping and assessing the impact of interventions in the region. This paper reports the first phase, a pilot study conducted in South Devon Healthcare NHS Foundation Trust’s Torbay Hospital, focusing on two interventions considered impactful, delivered at critical clinical and service decision points in patient care: (1) an advice and guidance (A&G) phone line on which a paediatrician is available for general practitioners (GPs) and the emergency department (ED) at all times and (2) SSPAU.

### Objective

The aim of this study was to describe and assess the impact of an A&G phone line and a SSPAU in reducing GP-referred attendances, admissions (including short-stay admissions) and length of stay of unplanned cases in Torbay Hospital.

## Methods

### Design

We used a 7-year series of routine observational data to assess the impact of the two interventions implemented in sequence in Torbay. Intervention specifications were collected through telephone interview with the clinical lead (RT) and operations manager (GS). Routinely collected daily data relating to attendance, admission and length of stay (outcomes) were collected for the time period April 2009–December 2015. We prespecified data definitions and coding through collaboration with the clinical network and the local Academic Health Sciences Network (see online Supplementary material).

10.1136/bmjpo-2017-000235.supp1 Supplementary file 1

### Setting

Torbay Hospital is a foundation trust, medium-sized district general hospital, with paediatric services comprising a 19 bed/cot inpatient ward, including a two bed high dependency unit and six bed adolescent unit. Staffing consists of 13 acute consultants, eight level 1 training grades plus six middle tier trainees. The study population was all children (<18 years old) in the catchment area, estimated at 27 740 (figure from Local Authority Joint Strategic Needs Assessment, 2015). Importantly, this population increases in the summer months, but no robust estimate exists for this increase or health service use. Admission rates at Torbay, in the face of a national increase, are relatively flat; despite this, the clinical team are still implementing strategies to reduce unplanned acute admissions.

### Intervention

A number of local interventions were mapped and two initiatives selected as the focus, based on anticipated impact: an A&G phone line, established in April 2014, on which a paediatrician is available for GPs and the ED at all times. At the commencement of the A&G phase, there was an increase from one to two consultants available for acute service provision. This increase was partly to enable more consultant input and partly to compensate for reduction in numbers of middle-grade paediatric staff owing to rota gaps. The purpose of A&G was to enable timely, robust communication with hospital-based paediatricians to agree most appropriate direction for unwell children. The phone line was a single phone held by a consultant (09:00–21:00 weekdays, 09:00–15:00 weekends) and middle-grade doctors outside of these hours. From November 2014, calls could result in: referrals to the newly established SSPAU to be seen that day (Monday–Friday) or where it was felt that immediate assessment was not required: A&G to GPs and parents enabling them to manage at home, sometimes with further review; booked review on SSPAU early the next day or booked into urgent (1–2 week) slots in consultant or registrar clinics. Calls were logged with an A&G clinic code and a summary placed with the patient notes.

The second intervention—a SSPAU—was established in November 2014, operating at full capacity immediately (five beds, one cubicle (SSPAU reduced the number of ward beds by two)). SSPAU was intended to be a place between primary care, ED and the paediatric ward to reduce admission to the ward of those not requiring lengthy care/review. The unit opened 09:00–20:00 weekdays, with last admission at 19:00. Those present at 21:00 either stayed late to complete care, were admitted to the ward or, if no beds, were kept in the SSPAU and counted as ‘overflow’. No patients were returned to ED but accepted referrals later than 19:00 were redirected to ED.

The unit was staffed by senior nursing staff (Band 6), healthcare assistants and additional consultant (taking total to 13) and targeted acutely unwell children (referred by GPs and/or ED) plus routine and review cases. SSPAU has not changed outpatient management of chronic conditions but acute deterioration would go to SSPAU. Children needing resuscitation on arrival and those being sent by ambulance all went to ED first. GPs could refer acute concerns directly via the A&G phone or less urgent concerns by letter or fax. The consultant responsible for the SSPAU was also the individual holding the A&G phone line. During implementation of these intervention, there were a number of changes to the GP landscape, which we are unable to account for due to a lack of robust data but remain important contextual factors.

### Primary outcomes

We analysed routine hospital data for four service parameters for children under 18 years:

1. GP-referred attendances;
2. Paediatric ward admissions;
3. Less than 1 day admissions;
4. Length of stay on paediatric ward/s.

The A&G phone line was evaluated on all four parameters. The SSPAU was evaluated for paediatric ward admissions, less than 1 day admissions and length of stay. We had no rationale for believing SSPAU had an impact on GP-referred attendances so we did not test this.

We originally planned to analyse 48 hours readmission, but were unable to as our reclassification of SSPAU admissions as attendances meant that robust comparison data could not be collected given local system constraints.

### Data source

Daily data were retrieved from local systems by a business intelligence specialist at the hospital (RR) and aggregated into monthly totals for analysis. We define an admission as *presence in the hospital at midnight*. Importantly, these are *paediatric ward* overnight admissions not simply hospital admissions (ie, SSPAU admissions are reclassified as attendances) and so are consistent preintervention and postintervention. Owing to collection method, admissions include elective and non-elective cases; however, electives were similar across all included years, both as a raw value and as a proportion of total admissions (range 17.2%–23.5%). Length of stay on the ward was measured in whole days and we distinguished between short stays (1 day or less) and other lengths of stay (2+ days), although we recognise others have defined this differently (eg, <2 days).^16^

### Data analysis

Outcomes were measured with two distinct time periods: preintervention and postintervention, for each intervention. To assess the impact of the A&G phone line (introduced April 2014), the period April 2014–October 2014 was compared with the same period April–October in the preceding years combined for which data were available (2009–2013). To assess the impact of the SSPAU (introduced November 2014), the period November 2014–October 2015 was compared with November–October from 2009 to 2012, excluding the period November 2013–October 2014, which was confounded by the opening of the A&G phone line. Thus, the respective impact of the A&G phone line and the effect of the bundled A&G and SSPAU was assessed. For each outcome, preintervention and postintervention monthly totals were summarised using means and SD. Two sample t-tests were used to compare the outcomes between the preintervention and postintervention phases. Results of these analyses are reported as estimated differences in means (postintervention−preintervention), with 95% CIs and p values.

### Hypotheses

We specified hypotheses following discussion with the clinical leads:

- A&G phone line: assessed in isolation. We anticipated a decrease in GP-referred attendances. We also anticipated a decrease in all admissions and a reduction in short-stay admissions.
- SSPAU: we anticipated a reduction in all admissions and short stay admissions.
- Combined, therefore, we anticipated a decrease in attendances, admissions and short-stay admissions.

### Ethics

As a service evaluation, R&D management approval was sought and obtained from the Hospital R&D department.

## Results

**Figure 1:**
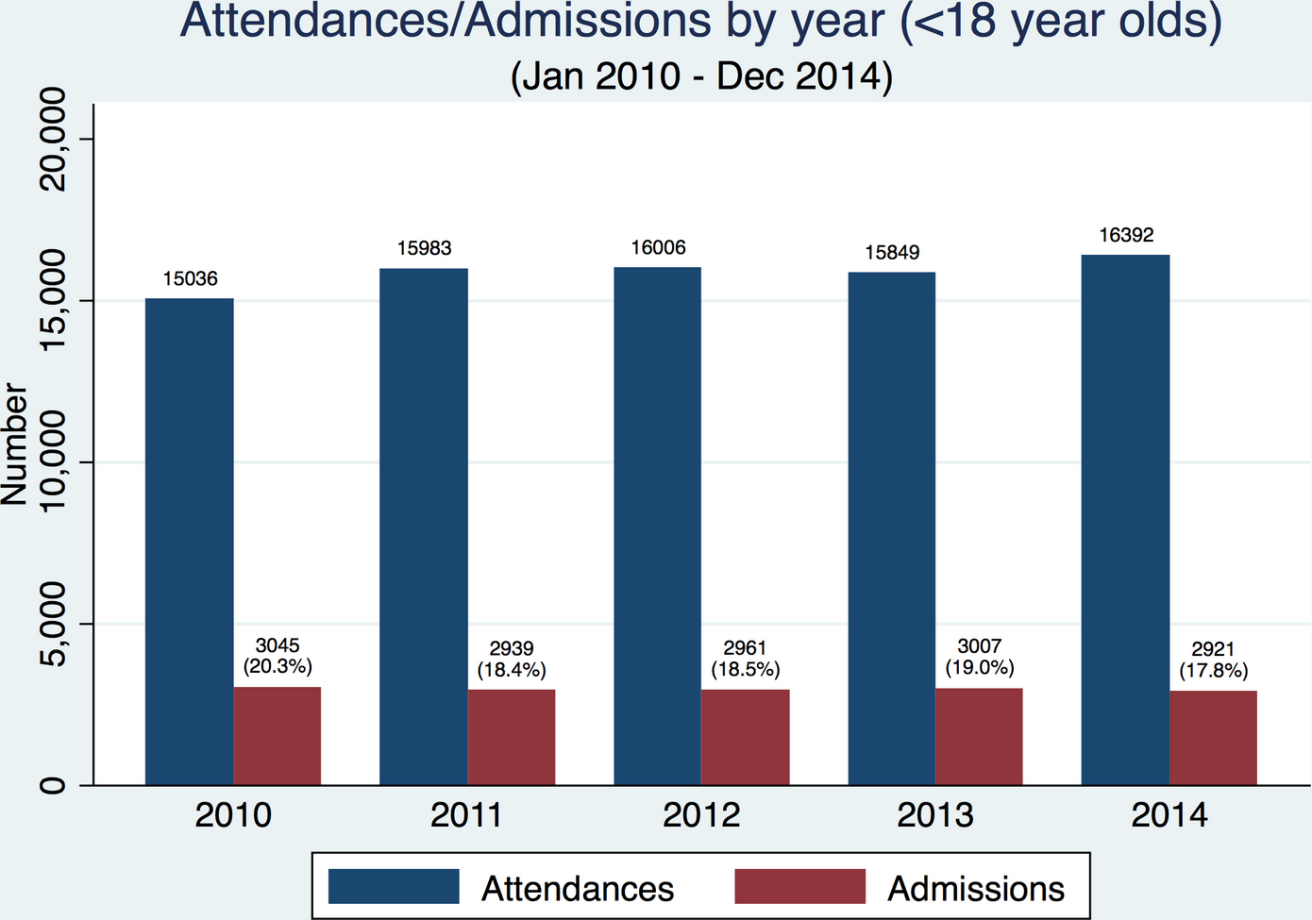
Total number of attendances and admissions at Torbay hospital each year, between January 2010 and December 2014.

Figure 1 shows the total number of attendances and admissions for the hospital between January 2010 and December 2014 (data not shown for incomplete years: 2009 and 2015). In the face of national increases,^1^ both the total number of attendances and admissions remain relatively constant in Torbay.

### Advice and guidance (A&G) phone line

**Figure 2:**
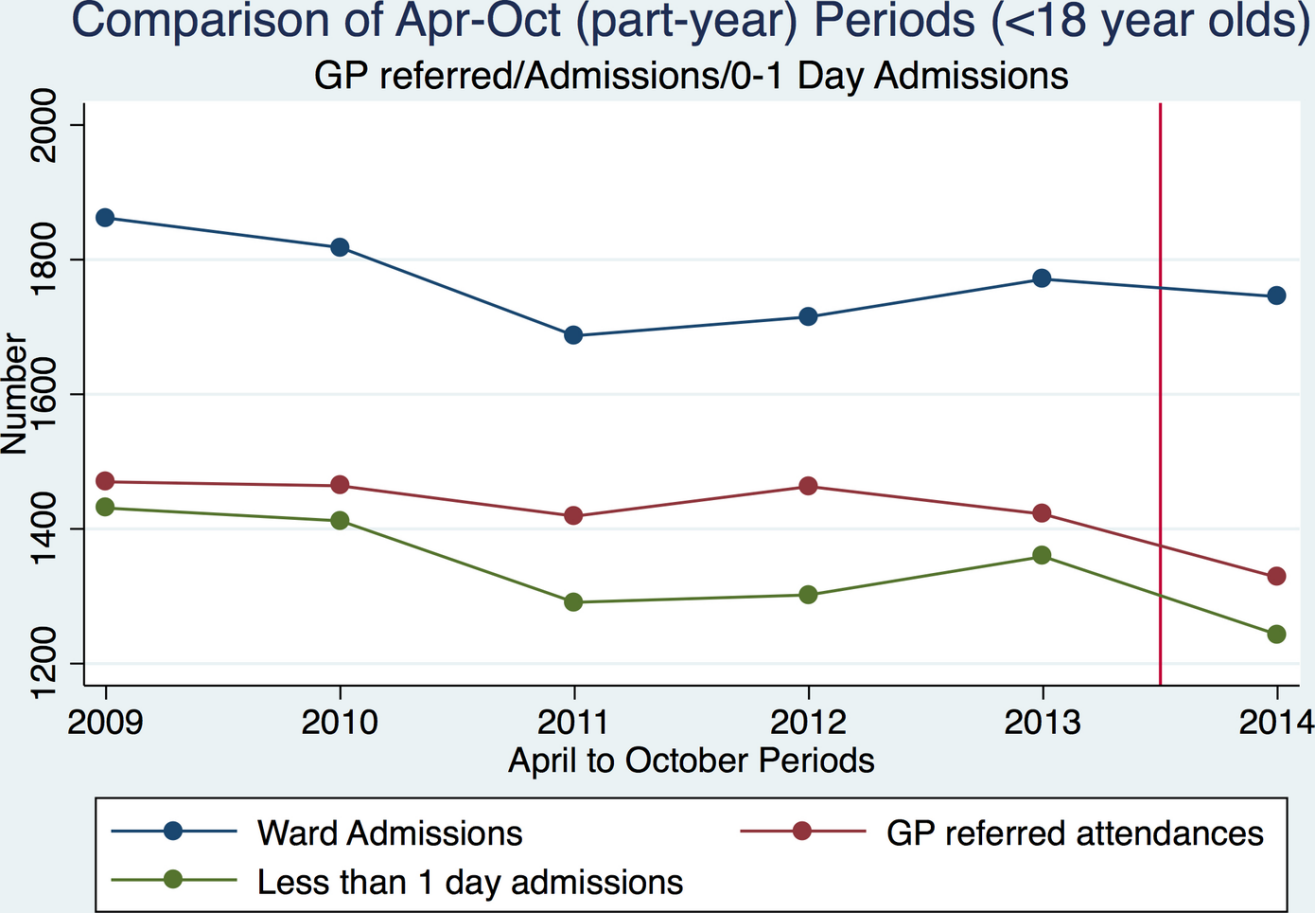
Total number of general practitioner (GP)-referred attendances, ward admissions and short stays at Torbay hospital, for each April–October part-year period between April 2009 and October 2014. Red vertical line indicates introduction of intervention.

We assessed the impact of the A&G phone line on GP-referred attendances, ward admissions, less than 1 day admissions and overall bed-days using the time periods specified. Figure 2 shows total GP-referred attendances, ward admissions and short stays for each April–October period.

There was little evidence of a change in monthly total GP-referred attendances postintervention (difference in means (post−pre) −17.1 (95% CI 5.6 to −39.8); p=0.1) or in monthly total ward admissions (difference in means (post−pre) −3.7 (95% CI 14.5 to −21.8); p=0.7) (table 1).

**Table 1:** Summary of main results

Intervention	Outcome	Preintervention mean (SD) monthly total	Postintervention mean (SD) monthly total	% Change in mean	Mean change post−pre (95% CI)	P values
		Apr–Oct 2009–2013	Apr–Oct 2014			
A&G	GP-referred attendances	206.8 (27.8)	189.7 (22.5)	−8.3%	−17.1 (5.6 to −39.8)	0.1
	Ward admissions	252.9 (22.6)	249.3 (15.8)	−1.5%	−3.7 (14.5 to −21.8)	0.7
	<1 day admissions	194.1 (20.3)	177.6 (14.2)	−8.5%	−16.6 (−0.2 to −32.9)	0.05
	Overall bed-days	341.9 (62.5)	414.4 (55.6)	21.2%	72.5 (124.0 to 21.0)	0.01
SSPAU		Nov–Oct 2009–2012	Nov–Oct 2014–2015			
	Ward admissions	248.0 (21.5)	213.4 (16.6)	−14.0%	−34.6 (-21.3 to −48.0)	0.0001
	<1 day admissions	186.6 (21.8)	164.9 (14.7)	−11.6%	−21.7 (-8.4 to −35.1)	0.002
	Overall bed-days	345.3 (60.4)	295.1 (52.5)	−14.5%	−50.2 (-12.1 to −88.3)	0.01

On average, monthly short-stay (less than 1 day) admissions reduced by 8.5% postintervention, from a mean monthly total of 194.1 (SD 20.3) to 177.6 (SD 14.2); difference in means −16.6 (95% CI −0.2 to −32.9); p=0.04 (table 1).

Monthly overall bed-days increased by 21.2% post-intervention, from a mean monthly total of 341.9 (SD 62.6) to 414.4 (SD 55.6); difference in means 72.5 (95% CI 21.0 to 124.0); p=0.01 (table 1).

### Short stay paediatric assessment unit

**Figure 3:**
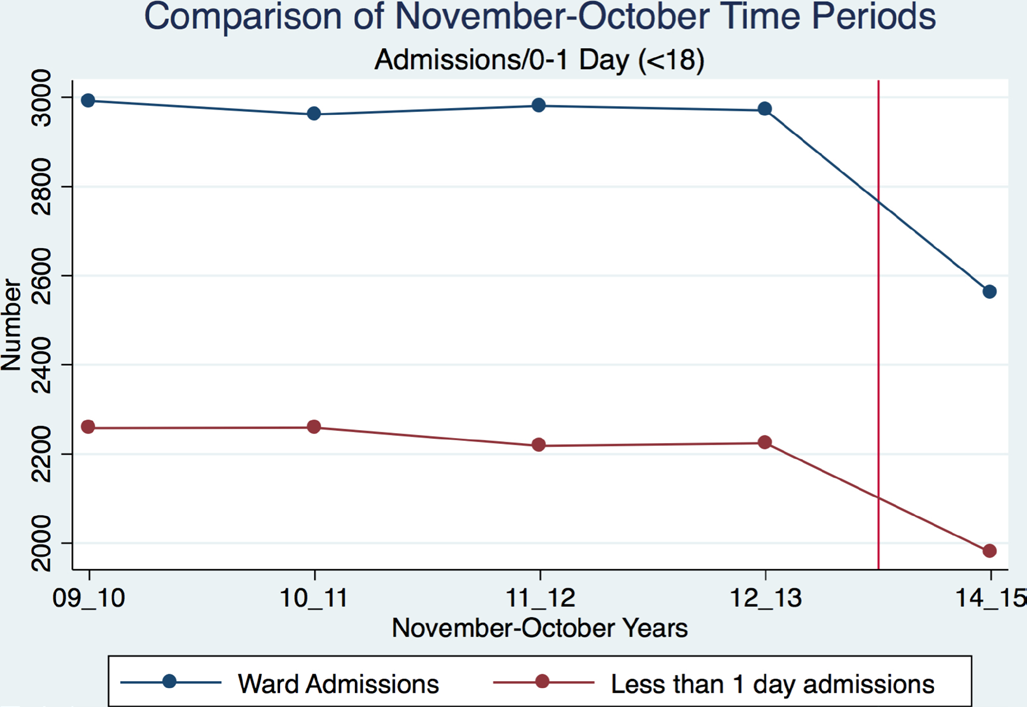
Total number of ward admissions and short stays at Torbay hospital, for each November–October period between November 2009 and October 2015. Red vertical line indicates introduction of intervention.

We assessed the introduction of the SSPAU in November 2014, therefore what is assessed is the bundling of A&G and SSPAU. Figure 3 presents total ward admissions and short stays for each November–October period.

There was strong evidence of a reduction in monthly admissions following the introduction of the SSPAU, from a mean monthly total of 248.0 (SD 21.5) to 213.4 (SD 16.6); difference in means (post–pre) −34.6 (95% CI −21.3 to −48.0); p=0.0001 (table 1).

On average, monthly short-stay (less than 1 day) admissions also reduced, by 11.6% postintervention, from a mean monthly total of 186.6 (SD 21.8) to 164.9 (SD 14.7); difference in means (post–pre) −21.7 (95% CI −8.4 to −35.1); p=0.002 (table 1).

Monthly overall bed-days reduced by 14.5% postintervention, from a mean monthly total of 345.3 (SD 60.4) to 295.1 (SD 52.5); difference in means (post−pre) −50.2 (95% CI −12.1 to −88.3); p=0.01 (table 1).

## Discussion

We anticipated decreased GP-referred attendances following the introduction of the A&G phone line. There was little evidence of a real change although the size of the reduction is consistent with the clinical view (RT) that around 10% of calls avoid admission through discussion. Importantly, the A&G line increased partnership working between paediatrics and primary care, enabling more responsive and flexible care, with GPs valuing consultant contact and the ability to manage acute illness through discussion.

We anticipated reduced admissions following introduction of the A&G line, of which there was some suggestion, but again little statistical evidence. This fits clinical description as, prior to the SSPAU, there was nowhere to manage cases other than ED or the ward. We anticipated short-stay admissions would reduce, with results indicating that this was significantly lowered postintervention.

With the introduction of SSPAU, we anticipated a reduction in admissions, less than 1 day admissions and bed-days. There was evidence that all of these outcomes reduced postintervention, and there are likely to be linked financial benefits, however, hospital-specific funding arrangements make robust assessments difficult; these interventions improve quality rather than simply reducing costs, with savings offset by the greater expense of providing additional consultant presence.

There was an increase in overall bed-days after the introduction of A&G, probably due to fluctuating numbers of long-stay cases, likely a direct impact of including mental health cases (something which is also likely to have had an impact on the decrease in ward admissions following the introduction of SSPAU).

These reductions in assessments in hospital care represent, we believe, not only an improvement for those individuals but also greater consultant involvement in assessment and management has reduced investigations and interventions. It is possible that some parents whose children were not admitted experienced increased anxiety managing them at home, but we believe that consultant review before discharge and safety netting allays most fears.

### Limitations/further research

Results presented here would usefully be broken down by injury/illness to assess the impact of true admissions against summer/visitor accidents. Additionally, amending the age profile to less than 10 years^16^ could reduce the impact of mental health cases on length of stay. It may be beneficial to examine these data by Big 6^6^ conditions; SSPAU may prevent asthma overnight stays, while A&G review may benefit fever cases.

## Conclusion

The introduction of an A&G phone line for GPs to contact paediatric consultants at Torbay hospital was associated with a decrease in less than 1 day admissions and an increase in overall bed-days. The later addition of a SSPAU alongside the A&G phone line was associated with a reduction in ward admissions, less than 1 day admissions and overall bed-days. Further work should explore these results by age, condition and injury/illness status.
