# Supplementary material for: Interventions for reducing unplanned paediatric admissions: an observational study in one hospital
Source: BMJ Paediatr Open. 2018 Mar 28;2(1):e000235. doi: 10.1136/bmjpo-2017-000235 (PMC5887827; doi:10.1136/bmjpo-2017-000235)
Supplement: Supplementary file 1 [file bmjpo-2017-000235supp001.pdf]

PAEDIATRIC ADMISSION PROJECT DATA DEFINITIONS

For the purpose of the Paediatric Admissions Project a child shall be defined as being of age less than 18 years.

Percentage of children attending ED admitted

Calculated as

- Number of children attending ED whose ACCIDENT AND EMERGENCY ATTENDANCE DISPOSAL is 01 - Admitted to a Hospital Bed /became a LODGED PATIENT of the same Health Care Provider

Divided by

- Total number of children attending ED

Percentage of children referred by GP who are admitted

Calculated as

- Number of children attending ED whose ACCIDENT AND EMERGENCY ATTENDANCE DISPOSAL is 01 - Admitted to a Hospital Bed /became a LODGED PATIENT of the same Health Care Provider and whose SOURCE OF REFERRAL FOR A and E is 00 - GENERAL MEDICAL PRACTITIONER

Divided by

- Total number of children attending ED whose SOURCE OF REFERRAL FOR A and E is 00 - GENERAL MEDICAL PRACTITIONER

Percentage of children referred by ED who are admitted

Calculated as

- Number of children attending ED whose ACCIDENT AND EMERGENCY ATTENDANCE DISPOSAL is 01 - Admitted to a Hospital Bed /became a LODGED PATIENT of the same Health Care Provider and whose SOURCE OF REFERRAL FOR A and E is **not** 00 - GENERAL MEDICAL PRACTITIONER

Divided by

- Total number of children attending ED whose SOURCE OF REFERRAL FOR A and E is **not** 00 - GENERAL MEDICAL PRACTITIONER

Total number of unplanned admissions

Calculated as

- Number of children whose ADMISSION METHOD is one of the following

21 Accident and emergency or dental casualty department of the Health Care Provider
22 GENERAL PRACTITIONER: after a request for immediate admission has been made direct to a Hospital Provider, i.e. not through a Bed bureau, by a GENERAL PRACTITIONER or deputy
23 Bed bureau
24 Consultant Clinic, of this or another Health Care Provider
25 Admission via Mental Health Crisis Resolution Team *
2A Accident and Emergency Department of another provider where the PATIENT had not been admitted **

2B Transfer of an admitted PATIENT from another Hospital Provider in an emergency **
2C Baby born at home as intended **
2D Other emergency admission **
28 Other means, examples are: ***
- admitted from the Accident and Emergency Department of another provider where they had not been admitted
- transfer of an admitted PATIENT from another Hospital Provider in an emergency
- baby born at home as intended

Length of stay of unplanned admissions

Calculated as

- Discharge Date minus Admission Date for all children whose ADMISSION METHOD is one of the following

21 Accident and emergency or dental casualty department of the Health Care Provider
22 GENERAL PRACTITIONER: after a request for immediate admission has been made direct to a Hospital Provider, i.e. not through a Bed bureau, by a GENERAL PRACTITIONER or deputy
23 Bed bureau
24 Consultant Clinic, of this or another Health Care Provider
25 Admission via Mental Health Crisis Resolution Team *
2A Accident and Emergency Department of another provider where the PATIENT had not been admitted **
2B Transfer of an admitted PATIENT from another Hospital Provider in an emergency **
2C Baby born at home as intended **
2D Other emergency admission **
28 Other means, examples are: ***
- admitted from the Accident and Emergency Department of another provider where they had not been admitted
- transfer of an admitted PATIENT from another Hospital Provider in an emergency
- baby born at home as intended

Age of child who is an unplanned admission

Calculated as

- AGE ON ADMISSION is derived as the number of completed years between the PERSON BIRTH DATE of the PATIENT and the START DATE (HOSPITAL PROVIDER SPELL)
- Into categories: (0-1/2-5/6-10/11-15/16-18)

Readmission rate of children seen in PAU

This will have to be undertaken locally by information teams within the Trusts

Calculated as

- The number of children seen in PAU who have an admission in the following 2 days whose ADMISSION METHOD is one of the following

21 Accident and emergency or dental casualty department of the Health Care Provider
22 GENERAL PRACTITIONER: after a request for immediate admission has been made direct to a Hospital Provider, i.e. not through a Bed bureau, by a GENERAL PRACTITIONER or deputy
23 Bed bureau
24 Consultant Clinic, of this or another Health Care Provider
25 Admission via Mental Health Crisis Resolution Team *
2A Accident and Emergency Department of another provider where the PATIENT had not been admitted **

2B Transfer of an admitted PATIENT from another Hospital Provider in an emergency **
2C Baby born at home as intended **
2D Other emergency admission **
28 Other means, examples are: ***
- admitted from the Accident and Emergency Department of another provider where they had not been admitted
- transfer of an admitted PATIENT from another Hospital Provider in an emergency
- baby born at home as intended

Divided by

- The number of children seen in PAU

Readmission rate of children admitted to a ward and discharged

This will have to be undertaken locally by information teams within the Trusts

Calculated as

- The number of children discharged from the hospital following an admission whose ADMISSION METHOD is in the following table and who have an admission in the following 2 days whose ADMISSION METHOD is in the following table

21 Accident and emergency or dental casualty department of the Health Care Provider
22 GENERAL PRACTITIONER: after a request for immediate admission has been made direct to a Hospital Provider, i.e. not through a Bed bureau, by a GENERAL PRACTITIONER or deputy
23 Bed bureau
24 Consultant Clinic, of this or another Health Care Provider
25 Admission via Mental Health Crisis Resolution Team *
2A Accident and Emergency Department of another provider where the PATIENT had not been admitted **
2B Transfer of an admitted PATIENT from another Hospital Provider in an emergency **
2C Baby born at home as intended **
2D Other emergency admission **
28 Other means, examples are: ***
- admitted from the Accident and Emergency Department of another provider where they had not been admitted
- transfer of an admitted PATIENT from another Hospital Provider in an emergency
- baby born at home as intended

Divided by

- The number of children discharged from the hospital following an admission whose ADMISSION METHOD is in the following table

21 Accident and emergency or dental casualty department of the Health Care Provider
22 GENERAL PRACTITIONER: after a request for immediate admission has been made direct to a Hospital Provider, i.e. not through a Bed bureau, by a GENERAL PRACTITIONER or deputy
23 Bed bureau
24 Consultant Clinic, of this or another Health Care Provider
25 Admission via Mental Health Crisis Resolution Team *
2A Accident and Emergency Department of another provider where the PATIENT had not been admitted **
2B Transfer of an admitted PATIENT from another Hospital Provider in an emergency **
2C Baby born at home as intended **
2D Other emergency admission **
28 Other means, examples are: ***
- admitted from the Accident and Emergency Department of another provider where they had

not been admitted
- transfer of an admitted PATIENT from another Hospital Provider in an emergency
- baby born at home as intended

ADMISSION METHOD

The method of admission to a Hospital Provider Spell.

Note: see ELECTIVE ADMISSION TYPE for a full definition of Elective Admission.

National Codes:

Elective Admission, when the DECISION TO ADMIT could be separated in time from the actual admission:

11	Waiting list
12	Booked
13	Planned

Note that this does not include a transfer from another Hospital Provider (see 81 below).

Emergency Admission, when admission is unpredictable and at short notice because of clinical need:

21	Accident and emergency or dental casualty department of the Health Care Provider
22	GENERAL PRACTITIONER: after a request for immediate admission has been made direct to a Hospital Provider, i.e. not through a Bed bureau, by a GENERAL PRACTITIONER or deputy
23	Bed bureau
24	Consultant Clinic, of this or another Health Care Provider
25	Admission via Mental Health Crisis Resolution Team *****
2A	Accident and Emergency Department of another provider where the PATIENT had not been admitted **
2B	Transfer of an admitted PATIENT from another Hospital Provider in an emergency **
2C	Baby born at home as intended **
2D	Other emergency admission **
28	Other means, examples are: ***<br>- admitted from the Accident and Emergency Department of another provider where they had not been admitted<br>- transfer of an admitted PATIENT from another Hospital Provider in an emergency<br>- baby born at home as intended

Maternity Admission, of a pregnant or recently pregnant woman to a maternity ward (including delivery facilities) except when the intention is to terminate the pregnancy

31	Admitted ante-partum
32	Admitted post-partum

Other Admission not specified above

82	The birth of a baby in this Health Care Provider
83	Baby born outside the Health Care Provider except when born at home as intended.
81	Transfer of any admitted PATIENT from other Hospital Provider other than in an emergency

Note: The classification has been listed in logical sequence rather than alphanumeric order.

***Note - National Code 25 *'Admission via Mental Health Crisis Resolution Team'* is only valid for use in the Mental Health and Learning Disabilities Data Set and the Commissioning Data Set schema version 6-2. This value is not permitted to flow in Commissioning Data Set schema version 6-1-1. Users of Commissioning Data Set version 6-1 must map National Code**

25 to another appropriate ADMISSION METHOD code for the purposes of flowing data through the Commissioning Data Set schema version 6-1-1.

**** Note - National Codes 2A, 2B, 2C and 2D have been introduced to replace National Code 28 '*Other means*'. Health Care Providers should use these codes locally for collection of data as soon as possible. However the codes are only enabled to flow in the Commissioning Data Set schema version 6-2, and this must NOT be done until Secondary Uses Service Release 13 (April 2013) as the Payment Grouper will not be updated to use the codes until then. Users should map locally collected new codes to old value 28 until notified by the Information Centre that they are accepted by the Payment Grouper. Records containing these codes prior to April 2013 will U group and will consequently not attract tariff. Also, users of Commissioning Data Set schema 6-1-1 should map these values to National Code 28 for submission in these data sets, until they are able to migrate to Commissioning Data Set version 6-2.**

***** Note - National Code 28 should only be used in Commissioning Data Set version 6-1, and in Commissioning Data Set version 6-2 until advised by the Health and Social Care Information Centre (as per note ** above). Otherwise it should only be used after April 2013 in Commissioning Data Set Version 6-2 where Health Care Providers have not yet updated their systems to collect National Codes 2A, 2B, 2C and 2D, or for submission of historical data which was not collected using the new National Codes. National Code 28 will be retired when Commissioning Data Set version 6-1 is superseded.**

ACCIDENT AND EMERGENCY ATTENDANCE DISPOSAL

A code to identify how an Accident and Emergency Attendance might end.

National Codes:

01	Admitted to a Hospital Bed /became a LODGED PATIENT of the same Health Care Provider
02	Discharged - follow up treatment to be provided by GENERAL PRACTITIONER
03	Discharged - did not require any follow up treatment
04	Referred to A&E Clinic
05	Referred to Fracture Clinic
06	Referred to other Out-Patient Clinic
07	Transferred to other Health Care Provider
10	Died in DEPARTMENT
11	Referred to other health CARE PROFESSIONAL
12	Left DEPARTMENT before being seen for treatment
13	Left DEPARTMENT having refused treatment
14	Other

For the Accident and Emergency Clinical Quality Indicators, further guidance on National Code '*Left DEPARTMENT before being seen for treatment*' is available on the Health and Social Care Information Centre website.

SOURCE OF REFERRAL FOR A and E

The source of referral of each Accident and Emergency Episode.

National Codes:

00	GENERAL MEDICAL PRACTITIONER
01	Self referral
02	Local Authority Social Services
03	Emergency services
04	Work
05	Educational Establishment
06	Police
07	Health Care Provider: same or other
08	Other
92	GENERAL DENTAL PRACTITIONER
93	Community Dental Service

References:

National Purchasing Unit for Dental Service Increment For Teaching (SIFT), 1996. Dental SIFT: Proposals for Minimum Data Set (MDS) requirements
